# Hesperetin loaded fucoidan selenium nanoparticles alleviate cisplatin induced acute kidney injury via antioxidant and cGAS STING pathway regulation

**DOI:** 10.1186/s11671-026-04871-5

**Published:** 2026-08-25

**Authors:** Lin Long, Yexi Zhang, Jun Xiao, Jianhua Zang

**Affiliations:** 1 Oncology Center I Department, Qingdao Hiser Hospital Affiliated of Qingdao University (Qingdao Traditional Chinese Medicine Hospital), Qingdao, 266000 China; 2 Rehabilitation Centre of Acupuncture and Massage, Qingdao Hiser Hospital Affiliated of Qingdao University (Qingdao Traditional Chinese Medicine Hospital), Qingdao, 266000 China

**Keywords:** Hesperetin, Selenium, Acute kidney injury, Cisplatin, cGAS-STING

## Abstract

**Purpose:**

To fabricate hesperetin-loaded fucoidan-selenium (Hes/Fu-Se) nanoparticles (NPs) for alleviating cisplatin-induced acute kidney injury (AKI), overcoming hesperetin’s low solubility and bioavailability and boosting antioxidant and anti-inflammatory efficacy.

**Methods:**

Hes/Fu-Se NPs were prepared by encapsulating hesperetin within fucoidan, and then functionalized selenium (Se) NPs. Nanoparticle physicochemical properties and antioxidant activity (FRAP, ABTS) were characterized. Cisplatin-stimulated HK-2 cells were used for in vitro detection of cell viability, apoptosis, ROS accumulation, mitochondrial function and cGAS-STING signaling. A cisplatin-induced AKI mouse model was adopted to test renal biochemical indexes, renal histopathology and renal injury-related protein expression.

**Results:**

Synthesized Hes/Fu-Se NPs exhibited uniform particle size, superior aqueous solubility and potent antioxidant activity. In vitro, the NPs restored HK-2 cell viability, mitigated apoptosis and ROS overproduction, and inhibited DNA damage and cGAS-STING cascade activation. In vivo, Hes/Fu-Se markedly reduced serum BUN and CRE, alleviated renal histological lesions and inflammatory infiltration, and downregulated the expression of cGAS, STING, γH2AX and TNF-α in kidney tissues.

**Conclusions:**

Hes/Fu-Se NPs relieve cisplatin-triggered AKI via amplified antioxidation, DNA damage restoration and cGAS-STING pathway suppression. This nanoformulation provides a promising nephroprotective strategy against chemotherapy-related renal toxicity for clinical translation.

**Graphical abstract:**

**Figure:**
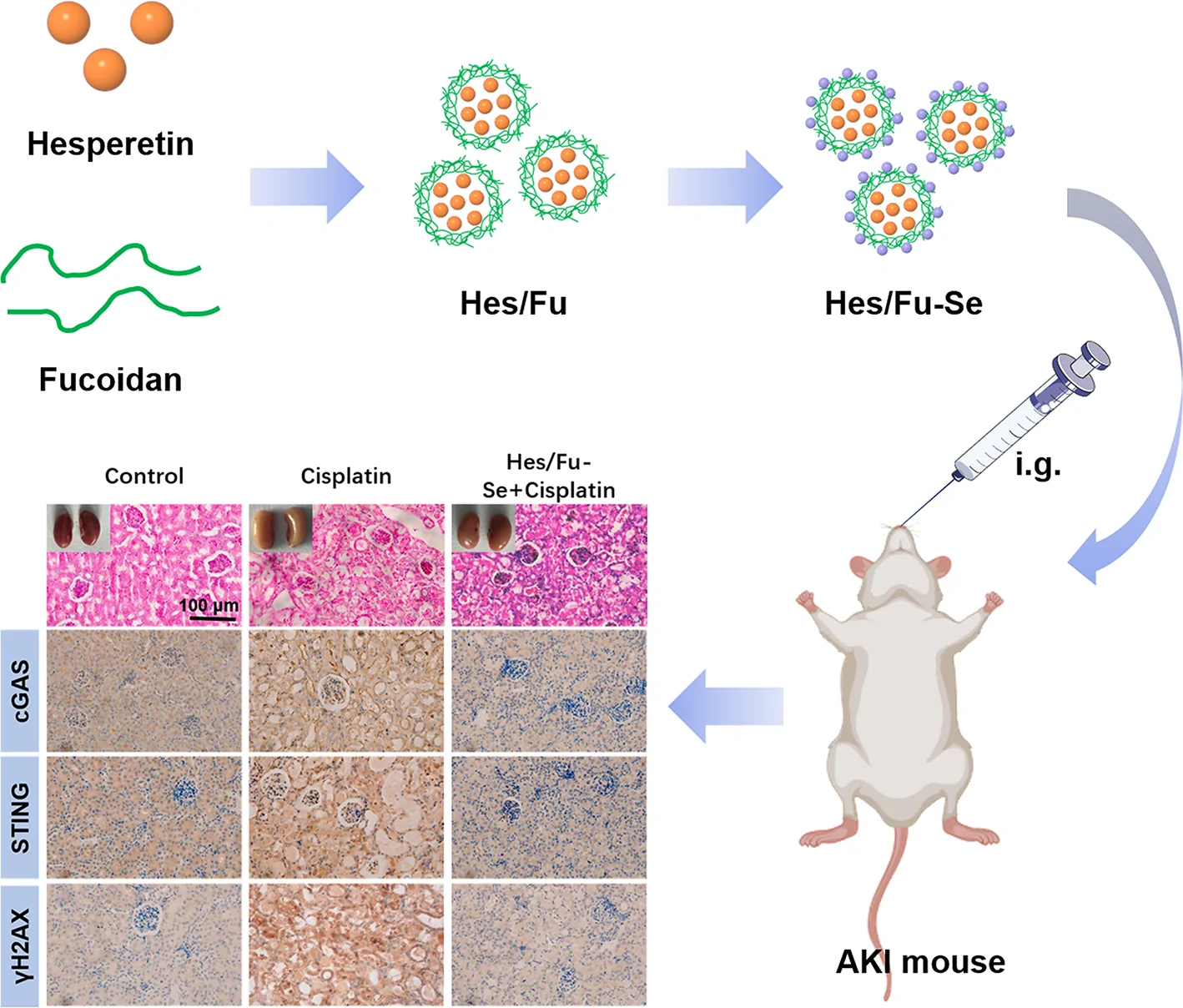

## Introduction

Acute kidney injury (AKI), a prevalent clinical syndrome, is marked by a sudden deterioration or complete loss of kidney function [1]. It has emerged as a growing global health concern, associated with elevated mortality rates, extended hospital stays, and an increased risk of developing chronic kidney disease [2]. The primary causes of AKI typically include hypovolemia, septic shock, and the use of nephrotoxic medications, with chemotherapeutic agents being a notable subset. Among these chemotherapeutic agents, cisplatin is one of the most common triggers of AKI [3].

Cisplatin, a well-recognized coordination compound also referred to as cis-diamminedichloroplatinum, belongs to the class of platinum-based chemotherapy drugs. It has established itself as a powerful therapeutic agent in the medical field, being employed to combat a diverse array of malignancies [4]. Owing to its remarkable anticancer activity, cisplatin has secured a prominent position as one of the first-line treatment options for numerous solid tumors, such as ovarian cancer, head and neck cancer, testicular cancer, and small-cell lung cancer [5, 6]. Cisplatin binds to the N7 position of purines, forming adducts that alter DNA activity and trigger apoptosis. However, the administration of cisplatin is not without its drawbacks. One of the most significant risks associated with this drug is nephrotoxicity. In a clinical environment, it has been observed that AKI occurs quite frequently, even when cisplatin is administered at low doses [7]. In fact, studies have shown that the incidence of AKI among patients undergoing cisplatin therapy reaches as high as 38% [3]. The mechanisms of nephrotoxicity involve proximal tubular injury, DNA damage, apoptosis, inflammation, oxidative stress, and vascular injury. Over the years, research has focused on finding an appropriate regimen to prevent cisplatin-related AKI. However, effective treatments remain scarce [8].

Natural products hold great promise as a potential solution for the prevention of AKI. A variety of natural compounds have emerged as effective agents, showing remarkable results in curbing cell death, oxidative stress, and inflammation [9]. For example, flavonoids, a class of widely-studied natural compounds, can protect the kidneys by reducing the accumulation of cisplatin in renal tubular cells or by alleviating the inflammatory response.

Hesperetin (3′, 5, 7-trihydroxy−4′-methoxyflavanone), a member of the flavonoid compound family, exhibits diverse biological activities including antioxidant, anti-inflammatory, and anti-apoptotic properties [10]. Numerous in vivo and in vitro studies have delved into the therapeutic potential of hesperetin across a wide spectrum of diseases [11]. For AKI therapy, hesperetin exerts renoprotection via modulating oxidative stress, suppressing inflammation and regulating apoptosis, making it a promising agent to alleviate AKI-related injury [12, 13]. However, despite its abundant natural sources, facile extraction and specific bioactivities, hesperetin suffers from prominent drawbacks. It possesses low aqueous solubility and instability within the gastrointestinal tract, which drastically impairs its oral absorption and bioavailability. Accordingly, constructing advanced delivery systems is critical to boost the in vivo bioavailability of hesperetin.

In this article, we developed a novel fucoidan-based nanocarrier system loaded with hesperetin and further modified with selenium (Hes/Fu-Se) NPs to alleviate AKI. The novelty of this system lies in its triple-component synergistic design, which integrates the stabilizing and bioactive functions of fucoidan, the antioxidant/anti-apoptotic effects of hesperetin, and the additional ROS-scavenging capacity of selenium—a combination not previously reported for AKI treatment. Benefiting from uniform particle size (~ 30.6 nm), superior water solubility, favorable hemocompatibility and feasible oral administration, the constructed Hes/Fu-Se NPs possess prominent practicability. Comprehensive in vitro and in vivo assays confirm that these NPs effectively mitigate cisplatin-induced AKI.

## Experimental

### Materials

Hesperetin, fucoidan, and anhydrous-grade sodium selenite were purchased from Shanghai Aladdin Biochemical Technology Co., Ltd. Polyvinylpyrrolidone (PVP-K17, average molecular weight of 8000) was obtained from Dalian Meilun Biochemical Co., Ltd. L-ascorbic acid and other chemical reagents were supplied by Sinopharm Chemical Reagent Co., Ltd.

### Characterization

Particle size distribution was measured at 25 °C using a NanoBrook Omni instrument (Brookhaven Instruments, Holtsville, NY, USA). UV–Vis absorption spectra were recorded on an ultraviolet spectrophotometer (TU-1950, Beijing Puxi General Instrument Co., Ltd.).

### Synthesis of Hes/Fu NPs

50 mg of PVP and 30 mg of hesperetin were dissolved in 2 mL of methanol separately and mixed. This mixture was then slowly added to a 10 mg/mL fucoidan solution (10 mL) under continuous stirring for 2 h, and yielding a stable dispersion of Hes/Fu NPs.

### Synthesis of Hes/Fu-Se NPs

To prepare Se-modified NPs, 500 μL of sodium selenite solution at varying concentrations (Na_2_SeO_3_-to-Hes/Fu mass ratios of 1:100, 1:50, 1:25, and 1:10) was introduced into 4 mL of the Hes/Fu dispersion and mixed thoroughly. Subsequently, 500 μL of L-ascorbic acid (80 mg/mL) was added and the mixture was stirred for 1 h to complete the reduction reaction, resulting in the formation of Hes/Fu-Se NPs.

### Determination of antioxidant activity in vitro

The in vitro antioxidant activity of Hes/Fu-Se was assessed using 2 methods: the ferric reducing antioxidant potential (FRAP) assay and the 2,2*’*-azino-bis (3-ethylbenzothiazoline−6-sulfonic acid) (ABTS) radical scavenging assay [14].

*FRAP assay*: The FRAP working reagent was prepared by combining 5 mL of 1 mM 2,4,6-tripyridyltriazine (dissolved in 40 mM HCl) with 5 mL of 2 mM FeCl₃ (prepared in sodium acetate buffer, pH 3.6). Then, 5 μL of 100-fold diluted Hes/Fu-Se and 180 μL of the FRAP working reagent were added to a 96-well plate. Absorbance changes at 593 nm were recorded immediately over time. Antioxidant activity was expressed as the concentration of the reduced product (Fe^2+^, mM).

*ABTS assay*: The ABTS radical cation (ABTS^+^) working solution was generated by mixing 7.4 mM ABTS with 2.6 mM K_2_S_2_O_8_ in a 1:1 volume ratio. The mixture was kept in the dark at room temperature overnight for complete oxidation. The resulting solution was diluted with PBS to an absorbance of 1.5 ± 0.5 at 734 nm. For the assay, 5 μL of diluted Hes/Fu-Se (100-fold) was mixed with 180 μL of ABTS^+^ working solution in a 96-well plate, and absorbance at 734 nm was monitored over time. The scavenging percentage was calculated as:

$${\mathrm{A}\mathrm{B}\mathrm{T}\mathrm{S}}^{+} \mathrm{s}\mathrm{c}\mathrm{a}\mathrm{v}\mathrm{e}\mathrm{n}\mathrm{g}\mathrm{i}\mathrm{n}\mathrm{g} (\mathrm{\%})=\frac{{\mathrm{A}}_{0}-{\mathrm{A}}_{\mathrm{t}}}{{\mathrm{A}}_{0}}\times 100\mathrm{\%}$$

where A_t_ represented the absorbance of the sample to be tested over time, and A_0_ represented the initial absorbance of the ABTS^+^ working solution.

### Cell viability

Human kidney-2 (HK-2) cells were obtained from the BeNa Culture Collection (Henan, China). Cells were maintained in RPMI-1640 medium supplemented with high glucose and 10% (v/v) fetal bovine serum (FBS) in a humidified incubator containing 5% CO_2_ at 37 °C.

For the viability test, HK-2 cells were seeded into 96-well plates at 4 × 10^3^ cells per well and allowed to adhere for 24 h. Thereafter, cells were exposed to 20 μM cisplatin, followed by treatment with various concentrations of Hes/Fu or Hes/Fu-Se 1:50 for an additional 24 h. Subsequently, 0.05 mg/mL MTT solution was added to each well and incubated for 4 h at 37 °C. The supernatant was carefully removed, and 150 μL of DMSO was added to dissolve the formazan crystals. The plates were shaken at 100 rpm for 20 min, and the absorbance at 490 nm was measured to determine cell viability.

### Assessment of cell morphology and apoptosis

HK-2 cells (1 × 10^5^ cells/well) were cultured in 24-well plates for 24 h, then treated with 20 μM cisplatin together with either Hes/Fu or Hes/Fu-Se 1:50 at a concentration of 10 μg/mL for 24 h. Morphological changes were observed and photographed using an optical microscope.

After treatment, cells were fixed with 4% paraformaldehyde for 15 min. Nuclear staining was performed using 500 μL of DAPI solution (10 μg/mL). Following washes, apoptotic nuclear alterations were visualized under a fluorescence microscope.

### Detection of reactive oxygen species (ROS)

HK-2 cells (1 × 10^5^ cells/well) were seeded in 24-well plates and cultured for 24 h. Cells were then exposed to 20 μM cisplatin along with 10 μg/mL Hes/Fu or Hes/Fu-Se for 24 h. Subsequently, cells were stained with 10 μM DCFH-DA for 30 min to evaluate ROS production. After three washes with PBS to remove excess dye, 500 μL of PBS was added, and fluorescence images were captured immediately using a fluorescence microscope.

### Rhodamine 123 staining for mitochondrial membrane potential

After the same treatment regimen as above, HK-2 cells were stained with 10 μM rhodamine 123 for 30 min. Unbound dye was removed by three PBS washes, and 500 μL of PBS was added. Fluorescence images were then acquired using a fluorescence microscope.

### Cellular immunofluorescence

HK-2 cells (2 × 10^9^ cells/well) were grown in 48-well plates for 24 h, then treated with 20 μM cisplatin together with 10 μg/mL Hes/Fu or Hes/Fu-Se for 24 h. Following treatment, cells were fixed with 200 μL of 4% paraformaldehyde for 15 min at room temperature, then blocked with 5% non-fat milk powder for 1 h to prevent non-specific binding. Primary antibodies (diluted appropriately in PBS) were applied and incubated overnight at 4 °C. After three washes with PBST, fluorescently labeled secondary antibodies (diluted as per manufacturer’s instructions) were added and incubated for 1 h at room temperature in the dark. Cells were then washed again with PBST, and nuclei were counterstained with DAPI for 5 min. Finally, PBS was added, and images were acquired using a fluorescence microscope.

### Cisplatin-induced AKI mouse model

Male Kunming mice (20–25 g) were purchased from Jinan Pengyue Animal Center (Jinan, China). Before model establishment, all mice were randomly divided into 6 groups (*n* = 7): (1) control (untreated), (2) cisplatin model, (3) cisplatin + 1 mg/kg Se NPs, (4) cisplatin + 50 mg/kg Hes/Fu, (5) cisplatin + 50 mg/kg Hes/Fu-Se 1:50 (low dose), and (6) cisplatin + 100 mg/kg Hes/Fu-Se 1:50 (high dose). AKI was induced by a single intraperitoneal injection of cisplatin (20 mg/kg). Test samples were administered orally once daily, starting 2 h before cisplatin injection and continuing for 3 consecutive days. On day 4, mice were euthanized by cervical dislocation under isoflurane anesthesia. Body and organ weights were recorded. Blood samples were collected for measurement of serum blood urea nitrogen (BUN) and creatinine (CRE). Kidneys were harvested for hematoxylin and eosin (H&E) staining and immunohistochemical analysis. Renal tissue was also used for RNA extraction to quantify the expression levels of cGAS, STING, γH2AX, and TNF-α via reverse transcription polymerase chain reaction (RT-PCR). During sample collection, biochemical detection, histological staining and quantitative image analysis, all operators were blinded to the group allocation of animal samples to avoid subjective evaluation bias. Primer sequences are listed in Table 1.

**Table 1:** Primers information

Gene	Forward primer	Reverse primer
m-TNF-α	ACAAGGCTGCCCCGACTAC	TGGGCTCATACCAGGGTTTG
m-GAPDH	GCCACCCAGAAGACTGTGGAT	GGAAGGCCATGCCAGTGA
m-STING	GGCTGGCCTGGTCATACTACA	CCCCACAGTCCAATGGAAAG
m-γH2AX	CATGTTCGTCATGGGTGTGAA	CCAGCAGCTTGTTGAGCTCCT
m-cGAS	AAGAGTTTCAAGAGCTGGATGCA	GGCACTCAAGAAAGAATGCTAACA

### Statistical analysis

All quantitative data were expressed as mean ± standard deviation (SD). Statistical comparisons among multiple groups were performed by one-way analysis of variance (ANOVA) and two-way ANOVA, followed by Tukey’s post hoc multiple comparison test to evaluate differences between every two groups. For each cellular experiment, n = 3 independent biological replicates were conducted; for animal in vivo assays, *n* = 7 mice per group (biological replicates). Statistical significance was defined as *p* < 0.05.

## Results and discussion

**Fig. 1:**
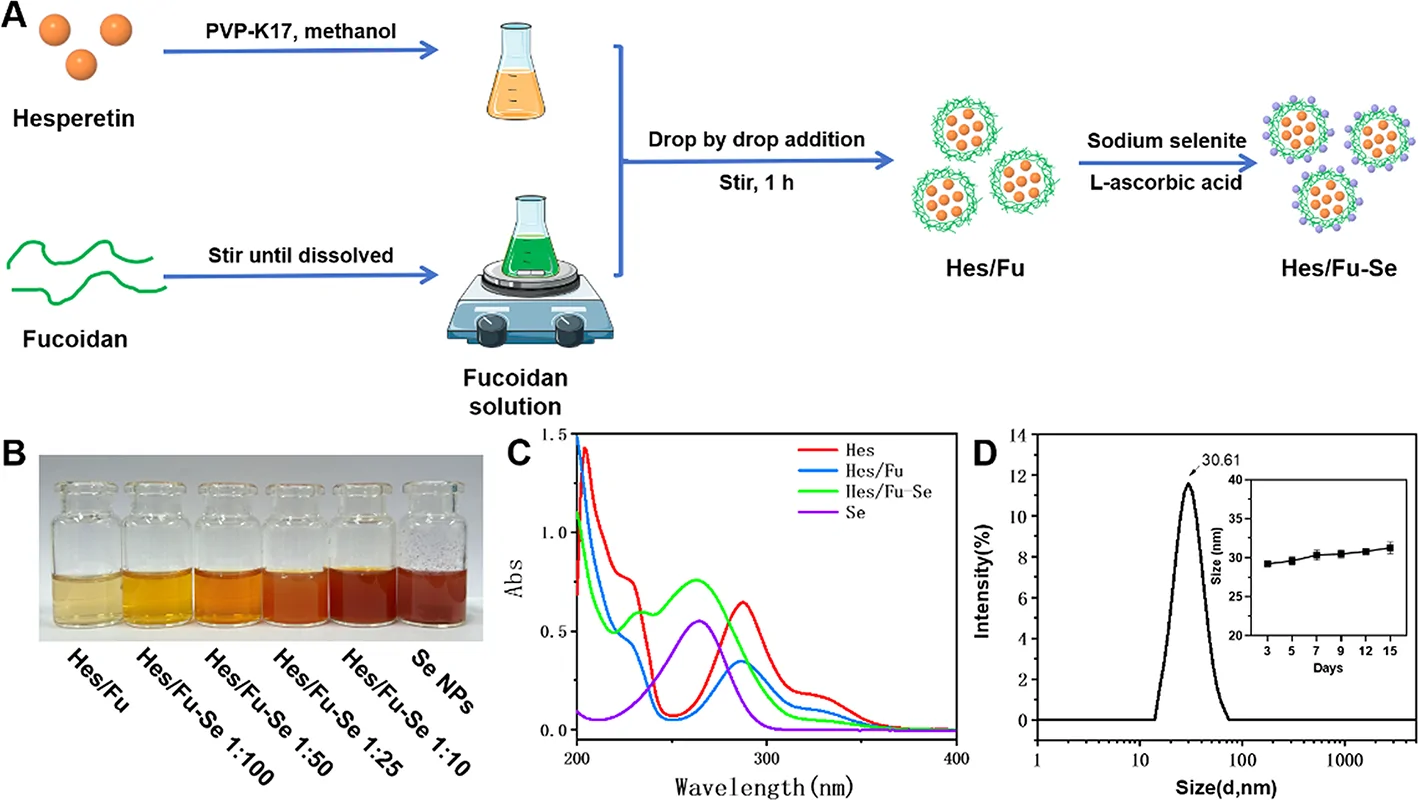
**A** Preparation of Hes/Fu and Hes/Fu-Se. **B** Appearance of Se NPs, Hes/Fu, and Hes/Fu-Se suspensions. **C** UV–vis spectra and **D** Granulometric distribution of Hes/Fu-Se. The inset image in (**D**) shows time-dependent size of Hes/Fu-Se within 15 days

Fucoidans, a type of sulfated polysaccharides, can be extracted from diverse species of brown algae [15]. They are already on the market as food or dietary supplements and are also used in cosmetics [15]. In the field of biomedicine, fucoidans have been proposed for the treatment of various diseases. This is because they have been proven to possess a variety of biological functions, such as antioxidant, antibacterial, anti-obesity, anti-allergy, anticancer, antiviral, anticoagulant, and antitumor capabilities [16]. When it comes to their role as drug carriers, fucoidans are able to not only stabilize drugs, which helps improve the drugs’ bioavailability, but also work in synergy with drugs to boost their effectiveness [17]. As shown in Fig. 1A, with the assistance of PVP-K17, fucoidans can uniformly load and disperse hesperetin. As a result, Hes/Fu NPs are formed. Subsequently, via the reduction reaction of sodium selenite, the Hes/Fu NPs undergo further modification with selenium (Se). This reaction leads to the formation of Hes/Fu-Se NPs. Se in the form of NPs can exert a protective effect against nephrotoxicity by inhibiting ROS-mediated apoptosis [18, 19]. This novel Hes/Fu-Se nanoparticle combines the biological activities of fucoidans, hesperetin, and Se NPs, potentially offering enhanced therapeutic effects due to the synergistic action of these components.

The Hes/Fu NPs display a consistent and transparent light-yellow color (Fig. 1B), indicates a well-formed and homogeneous NPs system. The dispersion of individual Se NPs presents a reddish-brown color (Fig. 1B) as well as a maximum absorption peak at 264 nm (Fig. 1C), a spectral feature associated with the electronic transitions within the Se NPs [20]. As for the Hes/Fu-Se NPs, as the loading of Se increases, the color of the dispersion gradually deepens (Fig. 1B). This change in color is a visual indication of the increasing Se content in the composite NPs. Additionally, similar to the individual Se NPs dispersion, the Hes/Fu-Se nanoparticle dispersion also exhibits a maximum absorption peak at 264 nm, suggesting the presence and influence of Se in the composite structure. Furthermore, Hes/Fu-Se NPs display a narrow size distribution, showing a peak diameter of 30.6 nm and a low PDI of 0.102 (Fig. 1D). The diameter of the Hes/Fu-Se NPs remained approximately unchanged throughout the entire storage period of 15 days, with only a slight and negligible upward shift, and no sharp size increase indicating particle aggregation. This well-defined size characteristic is crucial as it can influence the NPs’ physical and chemical properties, such as their reactivity and stability in various environments.

**Fig. 2:**
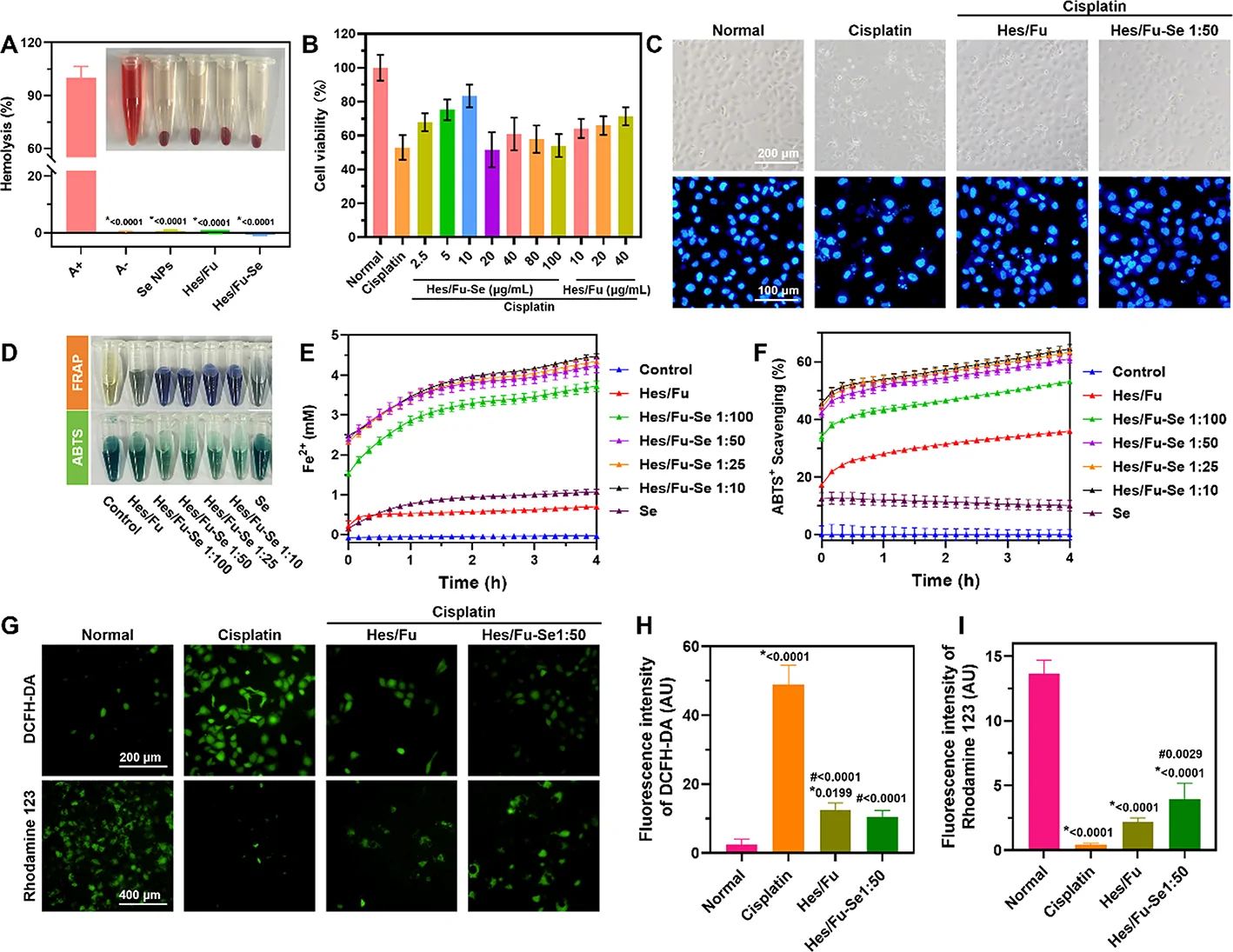
**A** Hemolysis of Se NPs, Hes/Fu, and Hes/Fu-Se, *p compared to the A^+^ group. **B** Cell viability of HK-2 cells after co culture with cisplatin, Hes/Fu, and Hes/Fu-Se for 24 h. **C** Cell morphology and DAPI staining of HK-2 cells after co culture with cisplatin, Hes/Fu, and Hes/Fu-Se for 24 h. **D**–**F** Antioxidant activity of Hes/Fu-Se confirmed by both the (**D**, **E**) FRAP assay and the (**D**, **F**) ABTS radical scavenging assay, **D** shows the photo of the reaction solutions. **G** DCFH-DA and Rhodamine 123 staining of HK-2 cells after co culture with cisplatin, Hes/Fu, and Hes/Fu-Se. **H**, **I** Fluorescence intensity calculated from (**G**), *p compared to the normal group, #*p* compared to the cisplatin group

To validate the biological activity of Hes/Fu-Se NPs, their hemolytic potential was initially evaluated. Hemolysis is a critical parameter to assess a material’s compatibility with blood components. As depicted in Fig. 2A, neither the individual Se NPs, nor the Hes/Fu NPs, nor the Hes/Fu-Se NPs exhibited hemolytic activity. Specifically, the hemolysis rates of all these NPs were far below 5%. This low hemolysis rate is a strong indicator of their excellent blood-compatibility, suggesting that these NPs are less likely to cause damage to red blood cells and are thus promising for potential in vivo applications.

The protective effect of Hes/Fu-Se NPs on cisplatin-treated cells is presented in Fig. 2B and 2C. As can be observed from Fig. 2B, cisplatin leads to a significant decrease in the viability of HK-2 cells. In contrast, both different concentrations of Hes/Fu-Se 1:50 NPs and Hes/Fu NPs can enhance cell viability to different extents. For the Hes/Fu-Se 1:50 NPs, at lower concentrations (2.5–10 μg/mL), the viability of treated cells gradually rises with increasing concentration, which indicates a concentration-dependent cell-protective effect. This implies that within a certain concentration range, the higher the concentration of the Hes/Fu-Se 1:50 NPs, the stronger the protection for the cells. However, when the concentration of Hes/Fu-Se 1:50 NPs reach a relatively high level (20–100 μg/mL), the protective effect does not continue to increase. Among the tested concentrations, the Hes/Fu-Se 1:50 NPs at a concentration of 10 μg/mL exhibit the highest cell-protective effect. Therefore, this concentration will be selected for subsequent experiments to further explore the potential of these NPs in protecting cells from cisplatin-induced damage.

As depicted in Fig. 2C, Hes/Fu-Se 1:50 NPs not only reduce cytotoxicity but also exert a significant influence on maintaining the morphology of cisplatin-treated cells and inhibiting apoptosis. Microscopic observations and DAPI staining results reveal that normal HK-2 cells possess a uniformly distributed cytoplasm, and their nuclei are circular or elliptical, positioned at the center of the cells. In contrast, the morphology of HK-2 cells treated with cisplatin undergoes remarkable alterations. Some of these cells exhibit fragmented nuclei. Hes/Fu NPs can mitigate cell apoptosis to a certain degree. In contrast, when HK-2 cells are treated with Hes/Fu-Se 1:50 NPs, their morphology is more similar to that of normal cells. This indicates that Hes/Fu-Se 1:50 has a protective effect on cell morphology, can reduce the incidence of cell apoptosis, and safeguard HK-2 cells from cisplatin-induced damage.

Both fucoidan and hesperetin possess antioxidant activity [21, 22]. Hes/Fu-Se NPs inherit the antioxidant properties of their individual components. The in vitro antioxidant activity of Hes/Fu-Se NPs has been confirmed by both the FRAP assay and the ABTS radical scavenging assay (Fig. 2D–F). Then, DCFH—DA, a commonly used fluorescent probe for detecting intracellular ROS levels [23], was employed to confirm the antioxidant activity of Hes/Fu-Se NPs at the cellular level. As depicted in Fig. 2G and H, in the presence of ROS within the cell, DCFH is oxidized to dichlorofluorescein, which emits strong fluorescence [24]. Hes/Fu-Se 1:50 NPs can significantly mitigate the fluorescence enhancement induced by cisplatin, thereby alleviating oxidative stress-induced damage to cells. Furthermore, Rhodamine 123 [25] was used to stain cells to confirm the effect of Hes/Fu-Se NPs on the cell health status. As depicted in Fig. 2G and I, cisplatin induced cell damage and the depolarization of mitochondrial membrane potential [26]. This led to the release of Rhodamine 123 from mitochondria and a decrease in fluorescence intensity. Meanwhile, Hes/Fu-Se 1:50 NPs could, to some extent, inhibit the changes in mitochondrial membrane potential and reduce cell apoptosis.

**Fig. 3:**
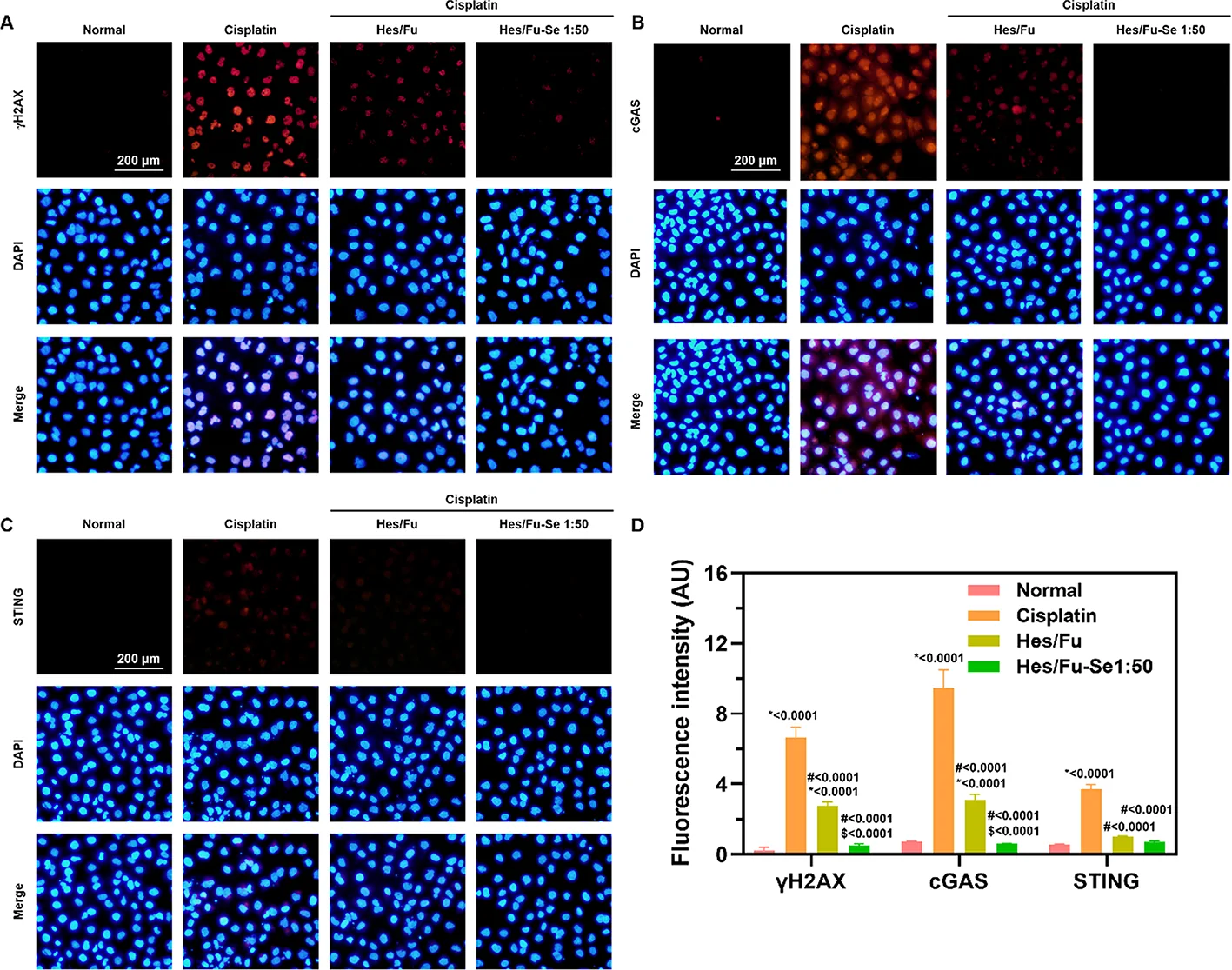
**A**–**C** Immunofluorescence analysis of the expression of **A** γH2AX, **B** cGAS, and **C** STING. **D** Fluorescence intensity calculated from (**A**–**C**), *p compared to the normal group, #*p* compared to the cisplatin group, $*p* compared to the Hes/Fu group

Hes/Fu-Se can also mitigate the DNA damage induced by cisplatin, as illustrated in Fig. 3A and D. γH2AX, a crucial molecular marker for DNA double-strand breaks [25], shows a rapid increase after HK-2 cells are treated with cisplatin. In contrast, Hes/Fu-Se 1:50 NPs can remarkably suppress this increase, suggesting that it has the potential to inhibit the occurrence of DNA double-strand breaks. Further immunofluorescence staining results in Fig. 3B–D indicated that the protective effect of Hes/Fu-Se 1:50 NPs is associated with the cGAS-STING pathway. Cisplatin can activate the cGAS-STING pathway [27]. By effectively repairing DNA damage, Hes/Fu-Se 1:50 NPs decrease the probability of DNA fragments leaking into the cytoplasm, thus reducing the activation risk of the cGAS-STING pathway.

**Fig. 4:**
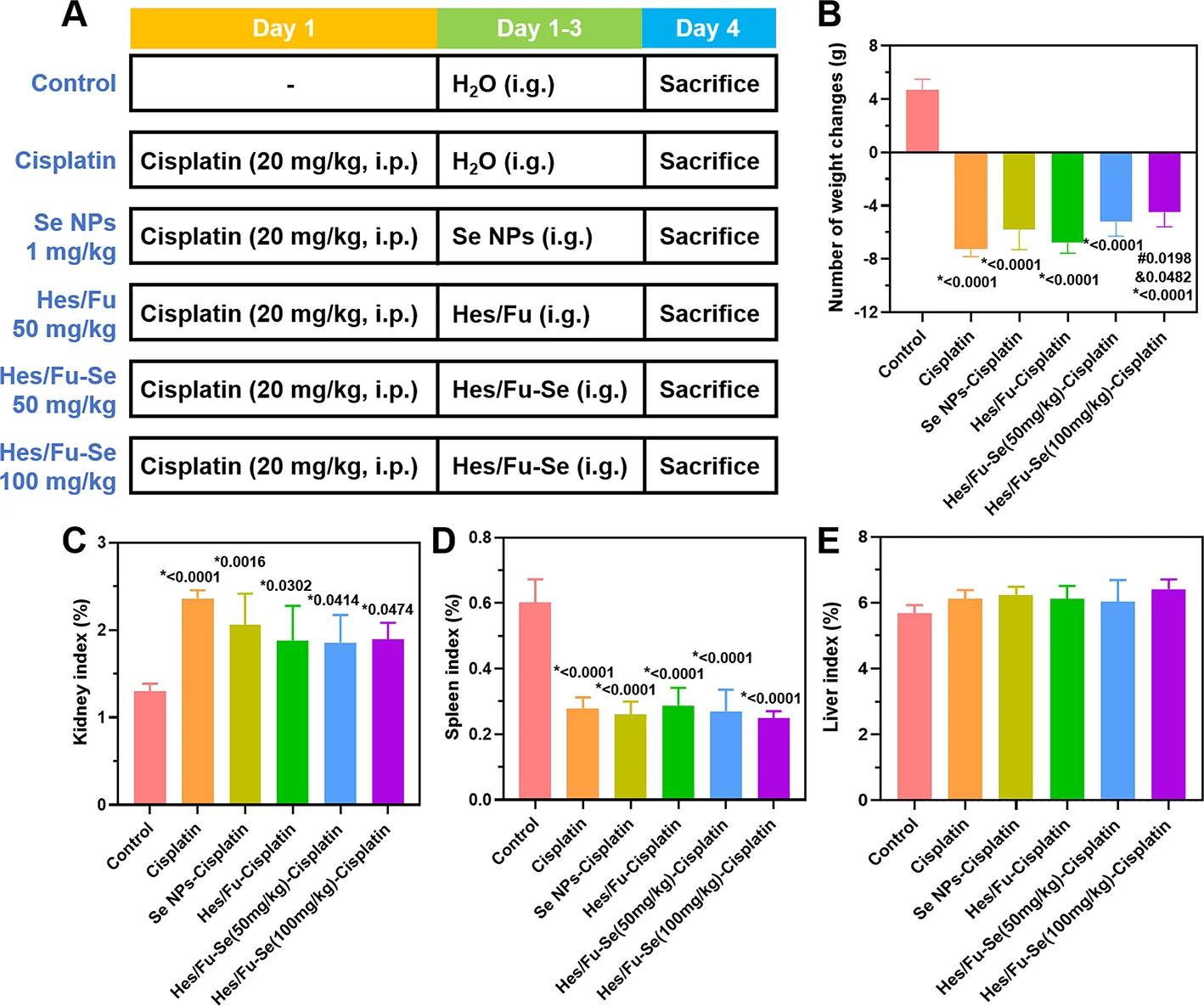
**A** Process design for animal study. **B** Weight changes, *p compared to the control group, #*p* compared to the cisplatin group, &*p* compared to the Hes/Fu group. **C**–**E** Organ index of **C** kidney, **D** spleen, and **E** liver, *p compared to the control group

The in vivo efficacy of Hes/Fu-Se NPs in alleviating nephrotoxicity was verified using a cisplatin-induced AKI model in mice [28]. Fig. 4A depicts the specific experimental groups of the animal study, as well as the dosage and administration route for each group. On the fourth day, the mice were euthanized, and their body weights and visceral organ weights were recorded in Fig. 4B–E. Cisplatin induced significant weight loss, an elevated kidney index, and a decreased spleen index in mice. This phenomenon can be attributed to the fact that during AKI, mice suffer from reduced food intake and metabolic disorders, which lead to weight loss (Fig. 4B). Additionally, the kidneys undergo a series of pathophysiological alterations, including the infiltration of inflammatory cells into the renal interstitium and the edema of kidney tissue, resulting in an increase in kidney weight (Fig. 4C). The spleen may shrink as a consequence of factors such as poor nutrition (Fig. 4D). Under certain conditions, Hes/Fu-Se 1:50 NPs, particularly at high doses, can ameliorate these abnormal changes. In addition, Hes/Fu-Se 1:50 NPs have no significant effect on the liver index of mice (Fig. 4E), indicating their good biocompatibility.

**Fig. 5:**
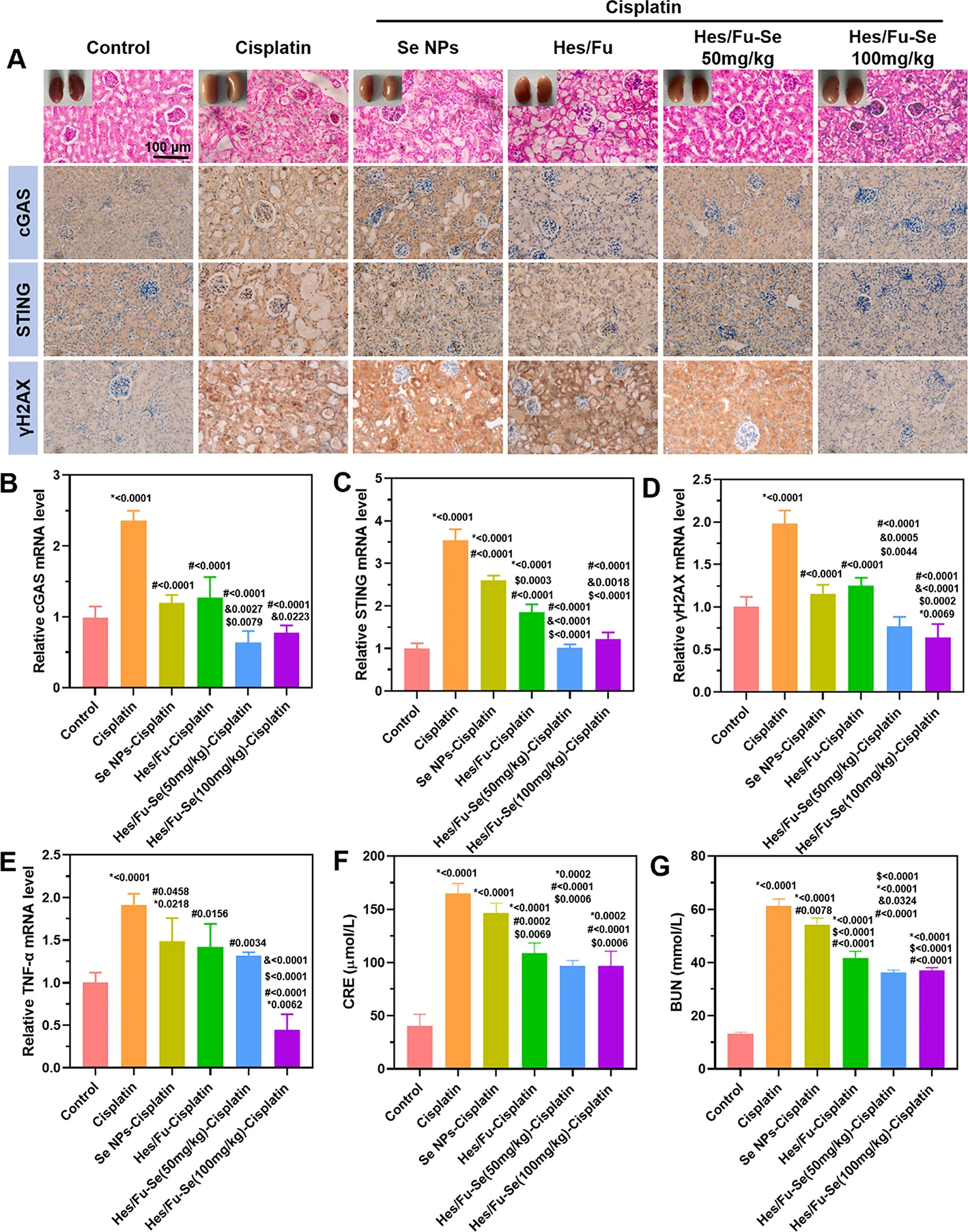
**A** H&E and immunohistochemical staining of kidney tissue. **B**–**E** The levels of **B** cGAS, **C** STING, **D** γH2AX, and **E** TNF-α in kidney tissue, **F**, **G** The levels of **F** CRE and **G** BUN in serum. *p compared to the control group, #*p* compared to the cisplatin group, $*p* compared to the Se NPs group, &*p* compared to the Hes/Fu group

The kidney photos (the inset pictures in Fig. 5A) reveal that the normal mouse kidneys have a uniform dark-red appearance with a smooth surface. In contrast, the kidneys of AKI mice exhibit ischemia, featuring a lighter color and an increased volume. HE pathological staining of the kidneys demonstrates significant infiltration of inflammatory cells and widening of the interstitial spaces in AKI mice (Fig. 5A). After the administration of Hes/Fu-Se 1:50 NPs, the kidney color gradually reverts to a shade close to the normal dark red, which indicates an improvement in renal blood perfusion. Moreover, the degree of kidney swelling decreases, and the kidney volume shrinks. Additionally, the infiltration of inflammatory cells in the renal interstitium is markedly reduced, and the edema is alleviated. Immunohistochemical staining of the kidney samples (Fig. 5A) and PCR results (Fig. 5B–D) confirmed that Hes/Fu-Se 1:50 NPs could alleviate kidney injury by inhibiting DNA damage and regulating the cGAS-STING pathway. DNA damage induced by cisplatin intake can activate the cGAS-STING pathway, leading to an up-regulation in the expression of pro-inflammatory cytokines, such as TNF-α (Fig. 5E). Hes/Fu-Se 1:50 NPs can inhibit the activation of the cGAS-STING pathway triggered by DNA damage, suppress the expression of pro-inflammatory cytokines, alleviate inflammation, and promote tissue repair.

Moreover, CRE and BUN in serum are biomarkers of kidney injury [29]. When mice develop AKI, the glomerular filtration function of the kidneys is impaired. As a result, the glomeruli cannot effectively filter CRE from the blood into the urine for excretion, which causes CRE to accumulate in the blood, leading to a significant increase in blood CRE levels. Simultaneously, the decrease in the glomerular filtration rate also reduces the excretion of BUN, thereby causing a significant rise in BUN levels. Hes/Fu-Se 1:50 NPs have the potential to alleviate kidney damage. By improving the glomerular filtration function, they can effectively promote the clearance of CRE and BUN from the blood. Consequently, this leads to a decrease in both blood CRE and BUN levels (Fig. 5F and G).

The development of NPs-based drug delivery systems for cisplatin-induced AKI represents a rapidly evolving field. AlBasher et al. demonstrated that Se NPs alone could protect against cisplatin nephrotoxicity by inhibiting ROS-mediated apoptosis in renal tissue [18]. Similarly, Li et al. reported that Se NPs synthesized using polysaccharide templates exhibited significant antioxidant activity and could attenuate cisplatin-induced oxidative damage [19]. On these bases, our Hes/Fu-Se system incorporates Se NPs as a functional component for drug encapsulation, expand its therapeutic potential. Secondly, we employ fucoidan—a natural sulfated polysaccharide with well-documented renal protective effects—as the primary carrier matrix. Fucoidan not only stabilizes the loaded hesperetin but also exerts intrinsic antioxidant, anti-inflammatory, and anti-apoptotic bioactivities, thereby generating synergistic therapeutic effects with Se NPs. Thirdly, flavonoid-encapsulated NPs are promising therapeutic candidates for AKI treatment. As a common dietary flavonoid, hesperetin boasts well-verified biosafety together with multifarious biological functions. Loading hesperetin into fucoidan mitigates its intrinsic limitations of weak aqueous solubility and inadequate bioavailability; meanwhile, integrated Se NPs further reinforce antioxidant capacity. Collectively, our composite delivery system exerts amplified therapeutic potency through triple synergistic functions: (1) fucoidan-supported particle stabilization and intrinsic bioactivities, (2) hesperetin-originated anti-oxidative and anti-inflammatory actions, (3) Se NP-mediated ROS elimination and renal protective effects. Such a multi-modal intervention can alleviate the intricate pathological lesions of cisplatin-evoked AKI, whose pathogenesis encompasses oxidative stress, DNA damage, inflammatory activation and mitochondrial impairment.

## Conclusion

Hesperetin-loaded fucoidan-selenium (Hes/Fu-Se) NPs possess uniform particle size, excellent water solubility and favorable biocompatibility, can effectively alleviate cisplatin-induced AKI in vitro and in vivo. Mechanistically, Hes/Fu-Se exerts synergistic nephroprotection via boosting antioxidant capacity, repairing cisplatin-triggered DNA damage, and suppressing the overactivated cGAS-STING inflammatory pathway. In cisplatin-challenged mice, oral Hes/Fu-Se lowers serum CRE and BUN, mitigates renal pathological damage and inflammatory infiltration, and recovers cisplatin-caused abnormal body weight and organ indexes. This triple-combination nanosystem provides a promising oral nephroprotective candidate against chemotherapy-related renal toxicity for clinical translation.

Several notable limitations exist in this work. First, only male Kunming mice were used for AKI modeling; gender-based differences in Hes/Fu-Se renoprotective effects remain unexamined. Second, we only conducted short-term observation within 4 days post cisplatin injection, and cannot confirm whether Hes/Fu-Se blocks the progression from AKI to chronic kidney disease. Third, the safety assessment is incomplete: only hemolysis and liver index were detected, while chronic Se accumulation toxicity and long-term off-target organ risks were not systematically evaluated.

Corresponding future research directions are proposed. First, female and aged mice will be introduced to explore gender- and age-dependent efficacy differences of Hes/Fu-Se. Second, long-term animal tracking will be performed to detect renal fibrosis markers and validate its inhibitory effect on AKI-to- chronic kidney disease transition. Third, repeated-dose toxicity tests will be carried out to comprehensively assess selenium deposition and systemic biosafety of the nanoformulation. Moreover, further formulation optimization and pharmacokinetic detection will help accelerate its clinical transformation.

## Data Availability

All experimental supporting data and procedures are available within this article.
